# Geochemical ceramic composition dataset using neutron activation and statistical analyses

**DOI:** 10.1016/j.dib.2023.109051

**Published:** 2023-03-08

**Authors:** Wael M. Badawy, Andrey.Yu. Dmitriev, Vladimir.Yu. Koval

**Affiliations:** a Frank Neutron Physics Laboratory, Joint Institute for Nuclear Research, 6, Joliot Curie str. 141980 Dubna, Russian Federation; b Egyptian Atomic Energy Authority (EAEA), Nuclear Research Center, Radiation Protection & Civil Defense Dept., 13759 Abu Zaabal, Egypt; c Institute of Archeology of the Russian Academy of Sciences, Moscow 119991, Russia

**Keywords:** Archaeological ceramics, Trace elements, INAA, Multivariate statistical analysis

## Abstract

These are comprehensive baseline data on the geochemical composition of archeological ceramics analyzed using instrumental neutron activation analysis (INAA). The data obtained support the research article conducted to evaluate the elemental composition of 70 sherds that were collected from different locations [1], [2], [3]. The mass fractions in wt% and in mg/kg of 39 oxides and elements were determined, respectively. Quality control of analytical measurements was carried out using different certified reference materials. Univariate and multivariate statistical analyses were performed. The common geochemical composition of the archeological pottery was used to decipher the provenance of ceramics and to establish reference groups based on various statistical approaches. For instance, hierarchical clustering (HC), linear discriminant analysis (LDA), principal component analysis (PCA) were used. The data was used to extract information about the important elements using machine learning (ML) methods. The obtained data show that chromium was the most important element and was used along with other elements as a fingerprint to distinguish the fragments. The chemical and statistical analyzes help to establish reference groups for medieval archeological pottery, which will be used in the future to classify and identify various unknown sherds. These reference groups serve as baseline data for determining where the fragments were made and are considered a reasonable judgment based on experimental data.

**Specifications Table**

**Table:** 

Subject	Archaeological Sciences

Specific subject area	Neutron activation and statistical analyses in life science
Type of data	Tables and figures
How data were acquired	The archaeological ceramics were subjected to INAA
Data format	Raw
Description of data collection	A total of 70 samples of archaeological ceramics were collected from various locations namely; Bolgar, Selitrennoe, Moscow, Rostislavl, Shamakhi, Nikolskoe, Tambovskaya, Ryazan, Smolensk, Bilyar, Djuketau, Donaurovskoe, Ostolopovskoe, and Tver. The samples were cleaned on-site and then transported to the laboratory for additional processing and preparation for INAA analysis.
Data source location	Samples were treated and analyzed in Joint Institute for Nuclear Research JINR, Joliot Curie st., 6, 141980 Dubna, Russia.
Data accessibility	The dataset is hosted by: Repository name: Mendeley repository Data identification number (permanent identifier, i.e., DOI number): DOI: 10.17632/whrn7bznfr.1 Direct link to the dataset: https://data.mendeley.com/datasets/whrn7bznfr/1
Related research articles	1. W. M. Badawy, A. Y. Dmitriev, V. Y. Koval, V. S. Smirnova, O. E. Chepurchenko, V. V. Lobachev, M. O. Belova, A. M. Galushko. Formation of reference groups for archaeological pottery using neutron activation and multivariate statistical analyses. Archaeometry. 64 (2022) - 1377-1393. https://doi.org/10.1111/arcm.12793

**Value of the data**

• The dataset provides the geochemical elemental analysis of archaeological ceramics. The dataset is of a great interest and importance that provides a comprehensive understanding of geochemistry of the studied ceramics.
• The dataset represents a comprehensive baseline data on the elemental composition of ceramics using NAA in Russia. The obtained dataset is useful for studying the provenance of ceramic shreds based on their geochemical content. The dataset can be used for comparison purposes to study the provenance of unknown fragments.
• The dataset could arouse the interest of researchers in various disciplines and it could be used as a supportive tool to the archaeologists, historians, physicists, chemists, statisticians, cultural heritage and in museums.
• The dataset can be considered as a background data and can be used for comparison purposes and decipher issues related to provenance and manufacturing of ceramics.

## 1 Objective

The current dataset was created to serve as baseline data and reference values for deciphering the origin of archeological pottery from various sites and fabrics. The geochemical data are an important tool to investigate the origin of the clay and raw materials used in ceramics. These data are used for comparison purposes with other types to investigate the extent to which they are typical in terms of elemental composition.

## 2 Data Description

**Fig. 1:**
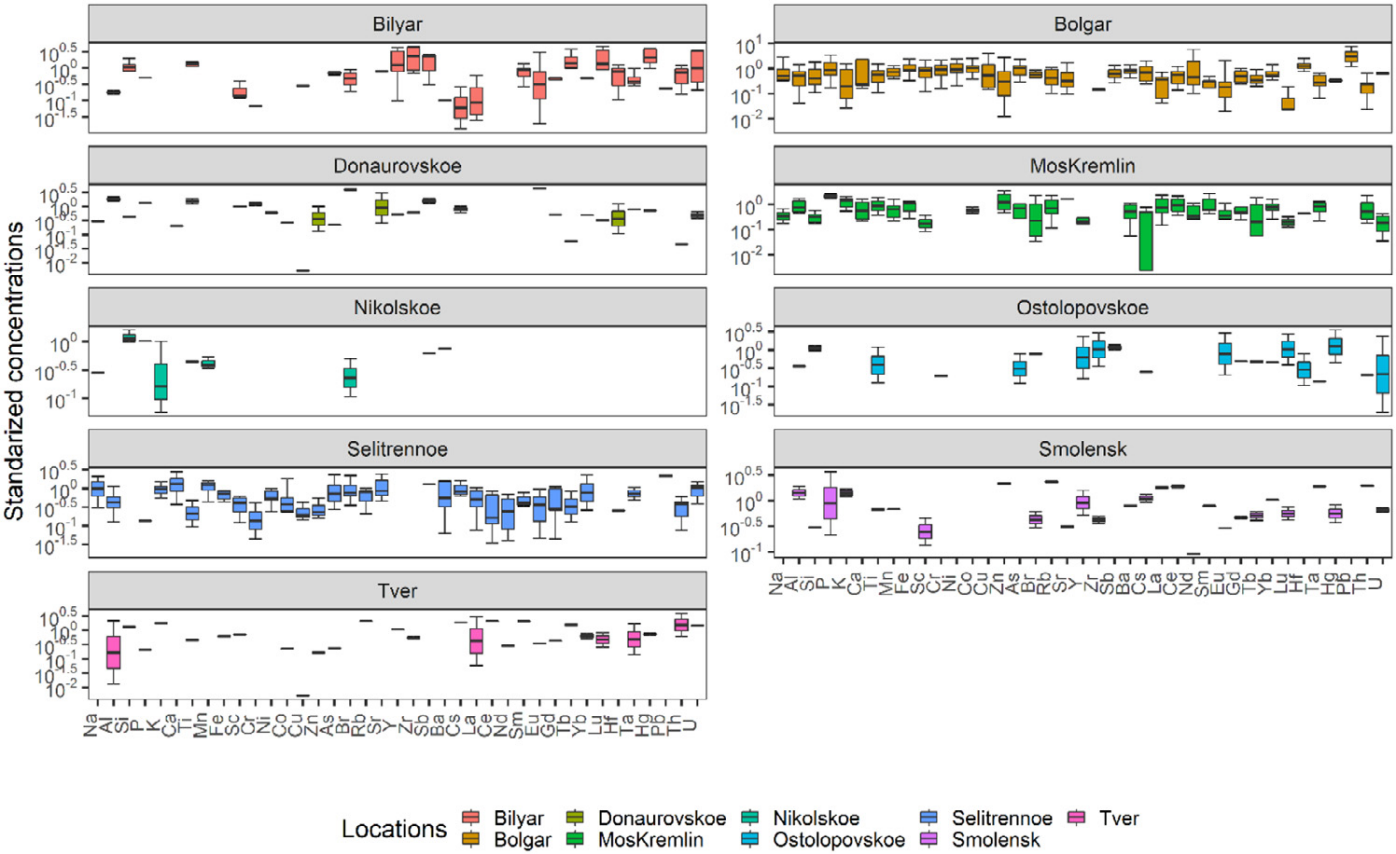
Boxplot illustrating the distribution patterns of the obtained data for each type of ceramics.

The elemental composition of a total of 70 fragments of archaeological potteries was published [3]. While the groups and the corresponding number of samples are published [4]. Furthermore, the obtained data were plotted and shown in Fig. 1. For better illustration, groups with a reduced number of samples were removed from the plot. The mass fractions of the obtained data were standardized prior to their plot to avoid the differences between the mass fractions of major and trace elements. Some The shreds were collected from 14 different cities and locations namely Bolgar, Selitrennoe settlement (hillfort), Moscow Kremlin (MosKremlin), Rostislavl, Shamakhi, Nikolskoe settlement (hillfort), Tambovskaya region, Ryazan, Smolensk, Bilyar, Djuketau, Donaurovskoe settlement (hillfort), Ostolopovskoe settlement (hillfort), and Tver. The elemental compositions in mg/kg of 39 trace elements were measured and the determined elements are Na, Al, Si, P, K, Ca, Ti, Mn, Fe, Sc, Cr, Ni, Co, Cu, Zn, As, Br, Rb, Sr, Y, Zr, Sb, Ba, Cs, La, Ce, Nd, Sm, Eu, Gd, Tb, Yb, Lu, Hf, Ta, Hg, Pb, Th, and U. The majority of the samples were collected from Bolgar, Selitrennoe settlement (hillfort), and Moscow Kremlin. Therefore, the reference groups were mainly created based on the data of the majority. The elemental concentrations were determined for the assemblages of medieval ceramics between X and XI centuries. Univariate and multivariate statistical data analysis were implemented. Hierarchical clustering (HC), linear discriminant analysis (LDA) and principal component analysis (PCA) were used. The findings demonstrate that Cr was important and may be used as a fingerprint, plotted alongside other elements to distinguish between different types of pottery based on geochemical data.

## 3 Experimental Design, Materials, and Methods

The ceramic samples were first cleaned of impurities in the field, the ceramics were transported to the laboratory for sample preparation. An angle grinder was used to separate small fragments from large ones. To avoid contamination, every ceramic sample had its outer layer removed by the angle grinder. The angle grinder 's disk was also cleaned after each use with ethyl alcohol to avoid cross-contamination of the samples. The samples were then individually washed with deionized water and soft brushes. After thorough cleaning, the samples were dried in a drying oven at 70°C for 2 hours. The samples were ground to a uniform particle size of 100 mesh using non-ferrous grinding tools (agate balls) to avoid contamination. The powder was dried to a constant weight at 104°C for 24 hours and then cooled in a desiccator. We used aliquots of 0.1 g of powdered ceramics and 0.1 g of certified reference materials. In addition to the certified reference materials, each sample was immediately packed in aluminum cups and placed in the desiccator with the other prepared samples. The preparation of the ceramic for NAA analysis has been described in detail elsewhere by Koval [1].

NAA was performed on the samples at FLNP, JINR by the group of GNAA. The samples were irradiated with an average power of 1.6 MW using the third channel of FLNP's pulsed nuclear reactor. The full characteristics of the channel were published by Koval [1]; Bulavin [5]. For 14 days, the ceramic samples were irradiated to thermal and resonance neutron fluxes of Φ_th_=7.88×10^11^ n/cm^2^ s and Φ_res_=2.43×10^11^ n/cm^2^ s, respectively. The samples are then stored for 3-5 days to decay to the induced radioactivity, at which point they can be handled. After repackaging the samples, the induced radioactivity for the first long-lived isotopes was determined using a gamma-ray spectrometer for a duration of 30 minutes. Similarly, the induced radioactivity of the second long-lived isotope was measured for 90 minutes after three weeks. The induced radioactivity of the obtained isotopes was measured at a resolution of 2.1 keV for the ^60^Co (1332 keV gamma energy line). Genie 2000 software was used to analyze the obtained spectra. The elemental composition of the ceramics was calculated in mg/kg (ppm) using a specially developed software by Dmitriev and Pavlov [6] and Dmitriev and Borzakov [7].

The NIST SRM 1633c - Coal Fly Ash, NIST SRM 2706 - Trace Elements in Soil, NIST SRM 1835 - Trace Elements in borate ore, NIST SRM 1632E - Trace Elements in Coal, NIST SRM 2709A – baseline Trace Element concentrations in San Joaquin Soil, and NIST SRM 2710A - Montana I Soil certified reference materials were used for quality control. The relative differences in concentrations range from 0% to 5%.

## Ethics Statements

This work did not involve studies with animals and humans.

## CRediT authorship contribution statement

**Wael M. Badawy:** Conceptualization, Data curation, Writing – original draft, Formal analysis, Visualization. **Andrey.Yu. Dmitriev:** Investigation, Resources, Methodology, Writing – review & editing. **Vladimir.Yu. Koval:** Supervision, Writing – review & editing.

## Declaration of Competing Interest

The authors declare that they have no known competing financial interests or personal relationships that could have appeared to influence the work reported in this paper

## Data Availability

Dataset of trace and major elements in archaeological ceramics (Original data) (Mendeley Data).
